# Young lungs cared enough? India's frontiers in diagnosing pediatric TB

**DOI:** 10.3389/fped.2025.1638167

**Published:** 2025-08-11

**Authors:** Silla Varghese Thomas, Priya Rajendran, Sivakumar Shanmugam

**Affiliations:** Department of Bacteriology, National Institute for Research in Tuberculosis- ICMR, Chennai, India

**Keywords:** children, pediatric tuberculosis, diagnosis, India, non-sputum-based testing, newer diagnostics

## Abstract

This review provides an insight into pediatric tuberculosis (TB) diagnosis in India. Significant challenges still exist in the accurate diagnosis of pediatric TB due to the paucibacillary status of the bacilli and the nonspecific clinical symptoms. Despite advancements in newer diagnostics that allow for rapid identification of TB and detection of drug resistance in children, their sensitivity is compromised due to these challenges. It is crucial to consider that children may not always expectorate sputum, further complicating the diagnostic process. Testing multiple samples, like aspirates, bronchoalveolar lavages, stool, urine, saliva, and swabs, may improve sensitivity. However, the efficacy of using these samples for pediatric TB diagnosis requires extensive research to validate their accuracy and reliability. This is crucial, especially in countries like India, which bears a high burden of TB cases, making the need for novel diagnostic approaches even more pressing. This need for innovative diagnostic approaches is particularly important in countries like India, which bears a high burden of TB cases. Collaborative efforts between researchers, healthcare providers, and policymakers are essential to drive innovation and progress toward achieving the END-TB goal. In this review, we have included studies and case reports published over a decade by utilizing scientific databases like PubMed, Scopus, and Google Scholar, and a set of key search terms including “pediatric TB in India”, and “pediatric TB diagnosis”.

## 1 Introduction

Tuberculosis (TB), an infectious bacterial disease caused by *Mycobacterium tuberculosi*s (MTB), serves as one of the leading causes of global childhood morbidity and mortality. India is considered to be one of the highest TB-burden countries and contributes about 26% of worldwide TB cases. In 2023, 12% (1.2–1.3 million) of the TB-infected population were children and young adolescents, with 15% of death cases reported worldwide (1). The high mortality seen in children is mainly due to a delay in diagnosis because of the non-specific findings from microbiological, clinical, and radiological features (2). The presentation of symptoms in children differs from that of adults, with an interval between infection and disease being comparatively shorter than the former. Significant challenges faced in pediatric TB diagnosis include paucibacillary status in sputum, the inability of children to expectorate sputum, non-specific clinical presentation, and the absence of a gold standard test (culture shows 40% sensitivity in pediatric TB) (3). Although the interpretation of chest radiographs is complex, pulmonary TB (PTB) diagnosis in children is mostly made based on clinical or radiological findings, as bacteriological confirmation may take time (4). Hence, depending on the resource settings and TB prevalence, diagnostic algorithms and approaches vary (5). Diagnosing TB, especially in children under 5, is crucial as by the year 2030, WHO End TB Strategy aims to decrease TB deaths by 90% and incidence rate by 80% (6). Hence, more advanced diagnostic techniques, including non-sputum-based specimens like oral swabs, stool, and urine, are paramount to achieving the End TB goal. Such novel diagnostic approaches are evaluated as multicentric studies by consortia like TB-SPEED and RaPAED (7). In this review, we aim to provide an update on pediatric TB from an Indian perspective and an overview of the currently available diagnostic methods. In addition, potential future diagnostic assays that could expedite the diagnosis of pediatric PTB are discussed.

## 2 Diagnostic methods for PTB

**Figure 1:**
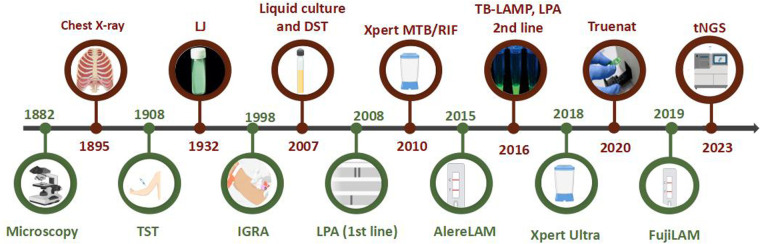
Timeline of TB diagnostic methods (images created using biorender.com).

The studies that report pediatric TB in India commonly involve smear microscopy, culture, Truenat, and Xpert testing for MTB detection. Smear microscopy is easy, quick, inexpensive, and one of the oldest methods recommended by WHO in the initial years of TB diagnosis for use in DOTS (Directly observed treatments, short course) (Figure 1) (8). However, with the given limitation of the paucibacillary state of pediatric TB and the high limit of detection (LOD) requirement for smear testing, the positivity rate is very low in children compared to adults and adolescents. Even though WHO recommends using molecular WHO recommended rapid diagnostic tests (mWRD) instead of smear microscopy due to their easy accessibility, the latter is still considered a primary-level diagnostic test for MTB detection (9). Mycobacterial culture is referred to as the gold standard for TB diagnosis. A study conducted in the year 2022 evaluated GeneXpert and liquid culture for the diagnosis of pediatric TB and reported additional MTB positives by Mycobacterial Growth Indicator Tube (MGIT) due to its long turnaround time (up to 42 days) (10). However, culture is regarded as an imperfect reference standard for pediatric TB (11). TB diagnosis has been easier after the involvement of automated nucleic acid amplification tests (NAATs). Country-wide studies conducted over a decade analyzing the sensitivity of these detection methods on pediatric TB diagnosis are discussed here.

### 2.1 Xpert MTB/RIF/Xpert ultra

Xpert testing is a cartridge-based nucleic acid amplification test (CB-NAAT) and serves as a preliminary diagnostic tool for MTB detection and its rifampicin resistance within 2 h. One large-scale presumptive pediatric TB study across India (Delhi, Chennai, Kolkata, and Hyderabad) reported 6.6% (6270/94,415) MTB positives by Xpert MTB/RIF (12). Other studies that have evaluated the performance of Xpert MTB/RIF in children from different types of respiratory samples are mentioned in Table 1. The updated version of the Xpert MTB/RIF assay is the Xpert Ultra, which runs on the same GeneXpert platform (FIND-Cepheid). Xpert MTB/RIF Ultra includes a result category called “trace call,” which indicates the presence of MTB at its lowest detection limit, which can be considered a positive finding in children. Xpert MTB/RIF Ultra assays have also shown a better detection rate in suspected TB cases, although the research done on this platform is limited. In addition to Xpert Ultra, WHO endorsed Xpert MTB/XDR in 2021, a new platform that detects resistance to isoniazid and 2nd line drugs (13). However, no studies have been published on Xpert MTB/XDR's use among the pediatric population.

**Table 1:** Studies demonstrating the specificity and sensitivity of the Xpert platform for MTB detection from respiratory samples.

S. no	Author and reference	Year	NAAT	Sample type	Sensitivity	Specificity
1.	Singh et al. (14)	2016	Xpert MTB/RIF	Respiratory samples	91.6%	86.8%
2.	Das et al. (15)	2019	Xpert MTB/RIF	Gastric aspirate, Cerebrospinal fluid (CSF), lymph node aspirate	98.04%	88.89%
3.	Saini et al. (16)	2018	Xpert MTB/RIF	Broncho Alveolar lavage (BAL)	87%	92%
4	Biduri et al. (17)	2024	Xpert MTB/RIF	BAL	78.9%	100%
5.	Singh et al. (18)	2021	Xpert MTB/RIF	Sputum, gastric aspirate, lymphnode aspirate	78.4%	83.9%
6.	Velayutham et al. (19)	2024	Xpert MTB/RIF	Sputum	68.5%	99%
7.	Singh et al. (20)	2015	Xpert MTB/RIF	Induced sputum	70%	94.3%
				Gastric aspirate	67.5%	89.6%
8.	Sharma et al. (21)	2020	Xpert MTB/RIF	Gastric aspirate	91.42%	98.45%
9.	Venkatesh et al. (22)	2021	Xpert MTB/RIF	Gastric aspirate	73.33%	93.6%
10.	Mishra et al. (23)	2023	Xpert MTB/RIF	Gastric aspirate	75%	94.5%

Diagnosis of TB using stool CBNAAT has now become an interest in children who find it difficult to expectorate sputum. In 2021, WHO recommended the use of Xpert Ultra for the diagnosis of pediatric TB using stool as the specimen (13). Few studies from India have also demonstrated the possibility of stool being considered an alternate sample for diagnosing TB in children (Table 2). Based on the research conducted so far, incorporating Xpert Ultra testing in stool can be recommended to improve TB diagnosis and management in children.

**Table 2:** Studies demonstrating the specificity and sensitivity of the Xpert platform for MTB detection from stool.

S. no.	Author and reference	Place	Year	Test	Reference standard	Sensitivity	Specificity
1.	Agarwal et al*.* (24)	Delhi	2021	Xpert MTB/RIF	MGIT of a respiratory sample	73%	97%
2.	Jayagandan et al*.* (25)	Bhopal	2022	Xpert MTB/RIF	Culture of respiratory sample	50%	100%
3.	Memon et al. (26)	Delhi	2018	Xpert MTB/RIF	MGIT of a respiratory sample	26%	100%
4.	Singhal et al*.* (27)	Agra	2024	Xpert MTB/RIF	Xpert and MGIT of a respiratory sample	57%	76%
5.	Torane et al*.* (28)	Maharashtra	2023	Xpert MTB/RIF	Xpert of a respiratory sample	100%	100%
6.	Suresh et al*.* (29)	Maharashtra	2020	Xpert MTB/RIF	LJ culture of gastric aspirate	75%	100%

### 2.2 Truenat

Truenat is a point-of-care (POC) test that uses the principle of real time Polymerase Chain Reaction (PCR). It is a chip-based assay using *nrdB* gene (Truenat MTB) for detecting MTB and *rpoB* gene (Truenat MTB-RIF) for detecting rifampicin resistance. The test usually takes 40–60 min. A comparison study by Urvashi et al. in 2023 reported the sensitivity of Truenat and Xpert MTB/RIF to be 58.7% and 56%, respectively (30). Another study published in 2023 reported the sensitivity of TrueNat and Xpert MTB/RIF to be 69% and 65%, respectively (31). Since only a few Indian studies have been published using truenat among the pediatric population, its utility in pediatric TB remains less explored.

### 2.3 TB-LAMP

WHO endorsed loop-mediated isothermal amplification assay (LAMP) for TB to replace smear microscopy in peripheral settings (32) to diagnose PTB or to follow up on smear-negative TB. TB-LAMP is based on an amplification process that uses four primers matching the target gene's six locations and a strand displacement reaction at 65°C for 15–60 min. This also makes the amplification process visible to the naked eye. The assay can be completed within 1 h.

Few studies conducted in India showed a sensitivity of 94.9% (33), 84% (34), and 75% (35), making it suitable for diagnosing PTB in children. The better sensitivity of LAMP makes it a suitable diagnostic method where no proper infrastructure is present to support Xpert.

### 2.4 Line probe assay (LPA)

LPA was approved by the WHO and is used under National Tuberculosis Elimination Programme (NTEP). It uses PCR and reverse hybridisation assay to simultaneously detect MTB, rifampicin resistance, high and low levels of isoniazid resistance. Mostly smear-positive samples or culture isolates are subjected to LPA and it takes around 48 h. This method was approved by WHO for detecting resistance to first-line (isoniazid and rifampicin) and second-line drugs (fluoroquinolones and injectables) (36). Although LPA is currently used for both adult and pediatric populations, research focusing exclusively on the diagnostic ability of LPA for pediatric TB detection is less. A study from Delhi in 2023, including 84 children, demonstrated the sensitivity of LPA to be 63.46% and specificity to be 100% (37). Another survey from Delhi used LPA to detect drug resistance in presumptive drug-resistant TB children, where among a total of 208 smear-positive or culture-positive, LPA had valid results for 198 children (38). In 2022, a study from Tamil Nadu demonstrated the ability of LPA to be used as a molecular method for detecting MTB from stool samples of healthy and confirmed TB children (39). Further research on LPA in pediatric TB detection and drug resistance is needed since the wait time for phenotypic drug susceptibility test (pDST) might lead to diagnostic delay and treatment.

## 3 Newer diagnostic methods

In the year 2023, WHO released “The Roadmap Towards Ending TB in Children and Adolescents,” which prioritizes research on child-specific diagnostics and treatments and also emphasizes the importance of TB in adolescents and pregnant and post-partum women (40). According to the report, despite the rise in the percentage of reported pediatric TB cases in 2022, a substantial treatment coverage gap was observed among young children. This underscores the critical need to develop innovative and prompt diagnostic methods to address this issue.

### 3.1 Lipoarabinomannan (LAM) assay

Using urine for detecting the LAM antigen of MTB has been used since 2015 (41). LAM is a glycolipid derived from MTB released into urine by either actively metabolizing or dying bacterial cells. The Alere Determine TB LAM Ag (Alere LAM) test was the first diagnostic kit developed to detect LAM in urine samples for TB patients. The test was recommended primarily for those with advanced HIV disease. In the case of children, since disseminated TB is common due to the non-specific initial presentation, LAM assays may have comparatively better sensitivity in children. The sensitivity of the LAM assay tends to rise in the later stages of tuberculosis, with a higher detection rate observed in malnourished children. This implies that malnutrition might lead to increased bacteremia, resulting in greater LAM excretion in urine and consequently enhancing the test's sensitivity (42).

A recent meta-analysis conducted in India in 2022 concluded that FujiLAM can be used as a POC for TB in HIV-negative children with suspected TB. Among microbiologically confirmed pediatric TB patients, pooled sensitivity and specificity were 52% and 90%, respectively (43).

### 3.2 TB infection diagnostic methods

QuantiFERON-TB Gold in the tube (QFT-GIT) or interferon-gamma (IFN-γ) assay (IGRA) is a diagnostic test used to assess cell-mediated immune response. Blood samples are collected in tubes coated with MTB antigen, and IFN-γ release is measured using the Enzyme Immunoassay (EIA) technique. In 2022, the WHO recommended using the tuberculin skin test (TST) or IGRA to diagnose TB infection (1).

According to the Latent TB infection (LTBI) guidelines from WHO in 2018, TST is recommended for children under the age of 5, while IGRA or a combination of IGRA and TST is preferred for children over 5 years old (44, 45). Another innovative skint test called Cy-TB was designed utilizing Early Secreted Antigen Target 6 (ESAT-6) and Culture Filtrate Protein 10 (CFP-10) antigens, the same components used in IGRA. It is administered and interpreted similarly to TST. This test also remains unaffected by Bacillus Calmette-Guerin (BCG) vaccination. It can be used as a Point Of Care (POC) test as well. The WHO endorsed Cy-TB in 2022 for its superior efficacy to TST (1). As per NTEP guidelines, these tests are conducted on individuals who have been excluded as having active TB (46). Although these tests have proven to be valuable supplemental diagnostics for TB infection in adults, their applicability in children, especially those under the age of 5, remains unexplored in India.

### 3.3 String test

The String Test (ST) was designed to diagnose enteric diseases for the isolation of *Giardia lamblia* and *Helicobacter pylori* from gastric samples. The test demonstrates complete (100%) sensitivity and specificity when evaluated by Xpert MTB/RIF and showed 87.5% sensitivity and 100% specificity compared to culture (47). This test is safe, cheap, and easily implemented without requiring hospitalization. It was also reported in a study from Peru that the pediatric population demonstrated good tolerance to ST compared to the collection of gastric aspirates, substantiating the potential of ST as an alternative diagnostic method (48). To date, there has been a lack of research conducted within the Indian pediatric population.

### 3.4 Swab tests

Oral swabs are one of the upfront and easy-to-obtain samples in the pediatric population. A study from Uttar Pradesh in 2021 demonstrated the use of buccal swabs in children using CB-NAAT to detect MTB. The study stated that patients with miliary TB were more likely to have buccal swabs positive for MTB (49). The use of swabs for the detection of MTB in India among children is not common. However, with a reported sensitivity of 5%–42% and specificity of 66%–100%, its use as an additional sample for diagnosing PTB in children needs to be considered (50).

### 3.5 Salivary biomarkers

Saliva is rich in immune response proteins. Cytokines in saliva and other protein biosignatures are promising biomarkers for screening TB (51), but no pediatric data are available. An Indian review article demonstrated the importance of analyzing not just a single biomarker but also the biosignatures that can help predict whether the child will develop or have TB (52). Being a child-friendly and readily available sample, saliva could also be considered an additional sample to help diagnose pediatric TB.

### 3.6 Bioaerosols

The first-ever study on bioaerosols was conducted in India, which seems to be a promising alternative for detecting pediatric TB (53). This pilot study suggested that detection based on RNA transcript has the potential to enhance TB diagnostic sensitivity in pediatric cases; however, additional studies are needed to confirm its clinical utility.

### 3.7 Next-generation sequencing (NGS)

Next-generation sequencing (NGS) is a technology that can be used either as whole-genome sequencing (WGS), targeted next-generation sequencing (tNGS), or metagenome sequencing (mNGS). While studies on WGS in adult TB patients are prevalent, data on pediatric TB are minimal. Since the studies in the adult population demonstrate the association between lineages and drug resistance, characterizing pediatric MTB isolates at the lineage level should also be given importance. Genotyping can enhance pediatric TB investigations by understanding the transmission dynamics. It can also be used to predict the drug resistance profile, as the turnaround time for pDST takes weeks. WGS is preferred chiefly over pDST in high-income, low-TB-burden settings. However, adopting WGS in low-income, high-TB-burden regions, like India, is the need of the hour. The requirement for specialized facilities with intricate workflows, proficient personnel, and data analysis proficiency impedes the technology's necessity (54). So far in India, no sequencing data is available on pediatric TB pulmonary isolates, and more research in this regard will pave newer research pathways.

### 3.8 Omics

In recent years, proteomics, metabolomics, and lipidomics have gained increasing interest. An Indian case-control study explored metabolic dysregulations in children with TB and identified panels of blood metabolites that showed good diagnostic accuracy for detecting MTB (55). Another research conducted in India revealed that pediatric TB patients exhibit distinct genetic profiles compared to adult tuberculosis patients (56). Although the field of omics provides interesting outcomes for pediatric TB, high cost and complexity limit the implementation of omics in routine diagnostics, especially in low-income countries. However, omics help enhance comprehension of host-pathogen interactions, particularly in biomarker exploration and the advancement of novel TB diagnostics. Hence, the possibility of using them in pediatric TB diagnosis must be explored.

## 4 Extrapulmonary tuberculosis (EPTB) diagnosis

**Figure 2:**
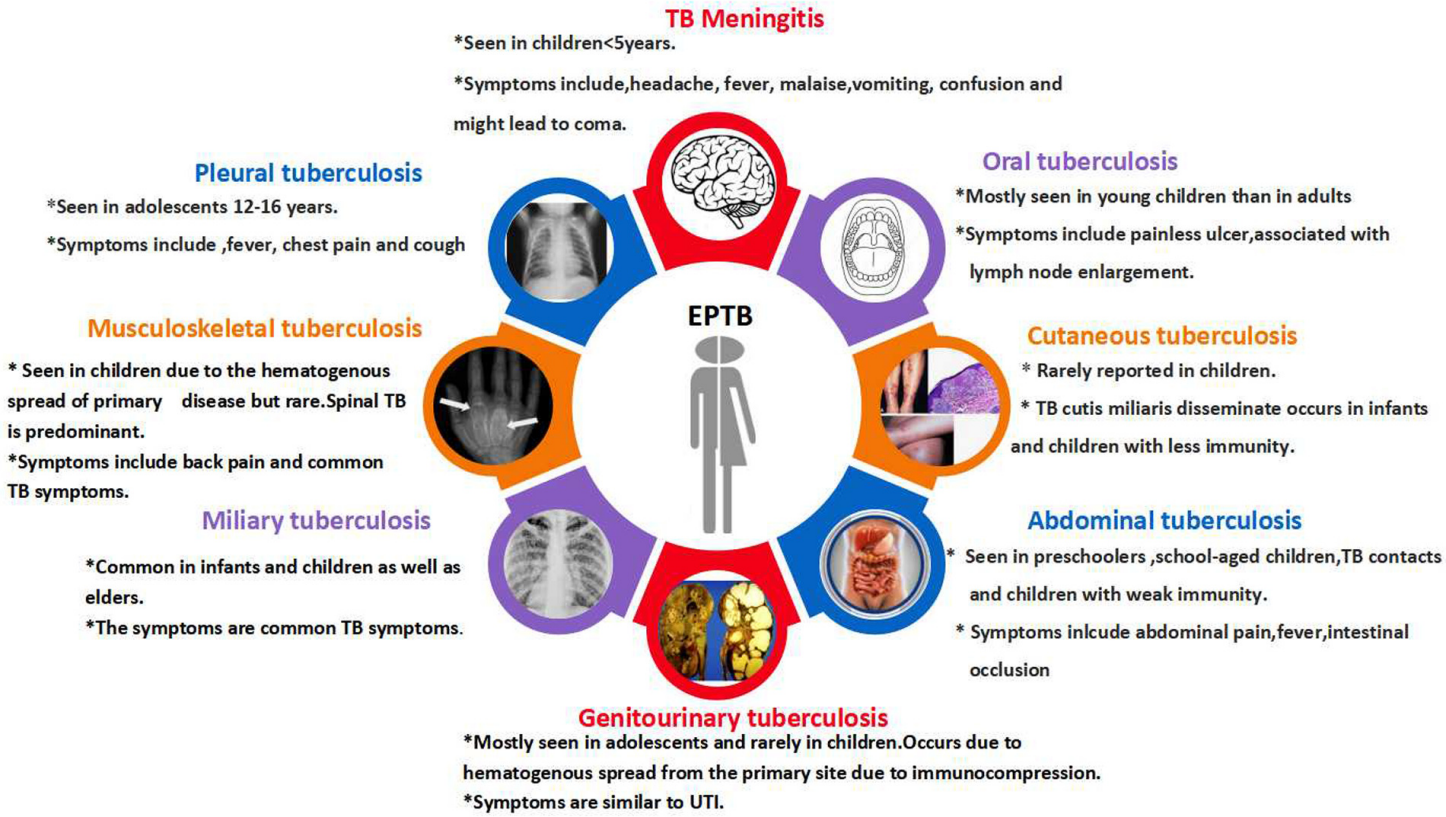
Different disease manifestations of EPTB.

The primary reason for EPTB's occurrence in children is lymphohematogenous dissemination. The Indian TB Report in 2023 documented the proportion of EPTB in children to be between 28% and 32% (57). The probability of acquiring the disease and the rate of dissemination increase with the decrease in patients' age (58). EPTB has different disease manifestations (Figure 2), and the selection of specific diagnostic tests depends on the type of clinical manifestation. Diagnostic methods for pulmonary TB are also utilized for EPTB, with the most common diagnostic test being NAAT (59).

Lymph node and tuberculous meningitis (TBM) are the most commonly observed forms of EPTB, and TBM is the most perilous form of EPTB. TBM presents with non-specific symptoms in the early stages and is usually diagnosed in the later stages of the illness, and most probably by then, the brain damage would have already occurred. Early diagnosis and management of TBM are essential, as a delay in diagnosis leads to neurological disorders and even death (60). It has been reported that TBM occurs in 0.3% of untreated TB infections in children, in the age group of 6 months to 4 years (61). A study conducted in India in 2021 utilized pyrosequencing in pediatric patients with TBM, demonstrating a notable sensitivity of 98.1%. Compared to the Xpert MTB/RIF assay and TB culture using MGIT, pyrosequencing of cerebrospinal fluid samples exhibited significantly superior sensitivity in diagnosing TBM (62). Furthermore, conducting additional research utilizing various sequencing methods within the pediatric demographic in India will be beneficial for prompt therapeutic decision-making.

The prevalence of other forms of EPTB, like cutaneous TB (CTB) in the pediatric population, was around 18%–56% of all skin TB in India. Scrofuloderma is reported more commonly in children, and this could be because of the consumption of raw milk, which may lead to infection by *Mycobacterium bovis* (63). A rare case of wrist TB was reported in a child, and this type of skeletal TB accounts for only <1% of all kinds of TB (64). Diagnosis of PTB itself is pretty challenging, and considering the symptoms of EPTB being non-specific makes the diagnosis even more tedious. EPTB contributes less to transmission than PTB and hence receives less attention. Large-scale studies focusing on this will help evaluate the risk of EPTB and resistance development. Apart from the studies published, there are also numerous case reports elucidating the significance of pediatric TB (Supplementary Table 1).

## 5 Conclusion

Closing the enduring gaps in the detection, treatment, and prevention of childhood tuberculosis is imperative to diminish the prevalence of pediatric TB (12%) and to achieve the END TB goal. The Roadmap towards Ending TB in children and adolescents emphasizes the pressing necessity for additional fundamental scientific research and implementation strategies to discover viable solutions for these persistent challenges. The past decade has witnessed heightened innovation across all TB spheres, particularly in pediatric TB. Implementing diagnosis via combinations of child-friendly samples like stool, urine, and oral swabs guarantees the reduction of the diagnostic gap seen in children. Child-friendly sample collection will help in the timely and accurate detection and contribute to TB control globally. Enhanced diagnostic accuracy, proper treatment protocols, improved nutritional awareness, and regular follow-ups would contribute to better outcomes, consequently resulting in the reduction of TB morbidity and mortality among children.
