# Supplementary material for: Young lungs cared enough? India's frontiers in diagnosing pediatric TB
Source: Front Pediatr. 2025 Aug 11;13:1638167. doi: 10.3389/fped.2025.1638167 (PMC12375632; doi:10.3389/fped.2025.1638167)
Supplement: Supplementary file 1 [file Table1.docx]

| **Type of EPTB** | **Place** | **Year** | **Diagnostic test** | **Case description** | **References** |
| --- | --- | --- | --- | --- | --- |
| Tuberculous meningitis | Puducherry | 2020 | MRI | An 11-month-old girl with regression of developmental milestones for over a month was admitted. A brain MRI revealed tubercles in the brain stem, which suggested tubercular meningitis. | 65 |
| Tuberculous otitis media | Delhi | 2020 | Smear microscopy | Four children <13 years were reported to have a discharging fistula, foul-smelling bilateral ear discharge, fever, headache, and decreased hearing. Microscopic examination revealed AFB bacilli. | 66 |
| Gingival TB | Delhi | 2019 | CBNAAT | A 13-year-old female was presented with painless swelling of the gingiva. Her sibling was previously treated for gastrointestinal TB. An incisional biopsy of the gingiva was carried out, and CBNAAT was positive for MTB. | 67 |
| Tubercular osteomyelitis | Delhi | 2022 | **Smear microscopy** | A seven-year-old female presented with swelling in the right eyelids and cheek for two months and was treated for orbital cellulitis, but was not responsive to treatment. AFB was detected in the pus smear, and the patient was diagnosed with tubercular osteomyelitis. | 68 |
| Cutaneous TB | Uttarakhand | 2021 | Histopathology | A 32-year-old woman and her 6-year-old were referred to the hospital due to the growth of lesions on the face. The boy had two well-defined, erythematous plaques on the nose and the left mandible. Histopathology examination revealed the presence of Langham cells, and the mother was TST positive. Based on this, Lupus vulgaris was confirmed, and ATT was initiated. | 69 |
| Liver TB | Haryana | 2021 | Smear microscopy | A 12-year-old girl was presented with abdominal pain with a history of fever and weight loss. On systemic examination, there was hepatomegaly. A computed tomography (CT) scan of the abdomen revealed a lesion involving the left and right lobes of the liver. A liver biopsy showed multiple epithelioid cell granulomas and was positive for AFB. | 70 |
| Pleural TB | Delhi | 2017 | Chest X-ray | An 11-year-old girl was presented with fever, breathlessness, cough, chest pain, and weight loss for around two months. Chest X-ray revealed pleural effusion, and TST was positive. Based on the Chest X-ray, pleural TB was confirmed, and ATT was initiated. | 71 |
| Abdominal TB | Uttar Pradesh | 2021 | Abdominal Xray | A 6-year-old boy was presented with intermittent fever for the past six months, low appetite, altered bowel habits, and painful abdominal swelling for the last three months. The abdomen radiograph showed diffuse calcified tubercles, and the patient was initiated on ATT. | 72 |
| Skeletal TB | Maharashtra | 2017 | MRI and smear microscopy | A 12-year-old girl presented with swelling of the left wrist joint with pain and restriction in movement. Magnetic resonance imaging of the left wrist showed infective arthritis, which was suggestive of TB. AFB was observed after ZN staining. | 73 |
| Miliary TB and disseminated TB | Chennai | 2021 | Imaging and culture | A 12-year-old was admitted to the hospital with a fever who had previously been diagnosed with lupus nephritis. Chest and abdomen imaging were suggestive of miliary TB. MTB was identified in bone marrow cultures, resulting in a diagnosis of disseminated TB. | 74 |

**Supplementary Table 1: Case reports on Pediatric EPTB**

**References**

65. Kasinathan A, Serane VK, Palanisamy S. Tuberculous meningitis manifesting with neuroregression in an eleven-month-old child. Indian J Tuberc. 2020 Jan;67(1):136-138. doi: 10.1016/j.ijtb.2020.01.001. Epub 2020 Jan 8.

66. Sebastian SK, Vijayan V, Kumar VB, Garg P. Tuberculous otitis media in children: Series of 4 cases. Int J Pediatr Otorhinolaryngol. 2020 Aug; 135:110118. doi: 10.1016/j.ijporl.2020.110118. Epub 2020 May 13.

67. Gupta S, Vats P, Jha A, Singh K, Ghosh S, Tandon S et al. Gingival manifestations of tuberculosis in pediatric patients: a series of 4 cases. Oral Surg Oral Med Oral Pathol Oral Radiol. 2019 Nov;128(5):508-514. doi: 10.1016/j.oooo.2019.01.005. Epub 2019 Jan 12.

68. Bhattacharya S, K Raina U, K Gupta S, Mishra M, Saini V, Kumar B. Tubercular Osteomyelitis of the Orbit Presenting as Periorbital Cellulitis. J Ophthalmic Vis Res. 2022 Jan 21;17(1):146-149. doi: 10.18502/jovr. v17i1.10181.

69. Arora K., Batra A., Dhanta A., Hazarika N. (2021). Lupus vulgaris in a mother and child. *BMJ case reports*, *14*(3), e240591. https://doi.org/10.1136/bcr-2020-240591

70. Kumar P, Sharma S, Banerjee A. Multicystic Hepatic Lesion: An Unusual Presentation of Extra-Pulmonary Tuberculosis in a Child. Indian Pediatr. 2021 May 15;58(5):485-486.

71. Yadav S, Rawal G. Primary extrapulmonary multidrug-resistant tuberculosis in an immunocompetent child presenting with pleural effusion. Transl Pediatr. 2017 Jan;6(1):72-75. doi: 10.21037/tp.2016.07.01.

72. Jaiswal R, Dubey DB. Child with a hard, calcified abdomen. IDCases. 2021 Jun 10;25: e01185. doi: 10.1016/j.idcr. 2021.e01185.

73. Shah MA, Shah I. Wrist swelling - Is it tuberculosis? J Family Med Prim Care. 2017 Oct-Dec;6(4):865-866. doi: 10.4103/jfmpc.jfmpc_200_17.

74. Janarthanan M, Antony T, Mohan R, Premkumar S, Pasupathy U. Disseminated tuberculosis with macrophage activation syndrome in a child with lupus nephritis. Sudan J Paediatr. 2021;21(2):190-194. doi: 10.24911/SJP.106-1614333951.
